# Characteristics of Friction Stir Welding of 3 mm thick ABS and PC thermoplastic polymers: An experimental approach

**DOI:** 10.1371/journal.pone.0322456

**Published:** 2025-05-08

**Authors:** Shaheer Ahmed Khan, Muhammad Sohail

**Affiliations:** Department of Materials Engineering, NED University of Engineering and Technology, Karachi, Pakistan; University of Vigo, SPAIN

## Abstract

The utilization of thermoplastics is extensively prevalent in modern industrial sectors owing to their distinctive mechanical features. Friction stir welding is recognized as a distinctive joining technology that addresses the weaknesses of heat-induced fusion welding. This friction-stirred solid-state welding technology can be effectively employed to join various difficult-to-weld polymeric materials. This paper examines the weldability of friction stir butt welding utilizing a cylindrical tapered threaded tool on a 3 mm thick Acrylonitrile Butadiene Styrene (ABS) and Polycarbonate (PC) polymers. The impact of tool rotational speed (800 and 1200 rpm) and tool traverse speed (10 mm/min to 50 mm/min) on the joint strength of welded samples has been analyzed. The maximum joint efficiency achieved is 52.71% for ABS while using a rotational speed of 1200 RPM and a traverse speed of 10 mm/min. For PC, the maximum joint efficiency is 54% with a rotational speed of 800 RPM and a traverse speed of 40 mm/min. The joint efficiency of polymer is significantly improved as a result of the effective heat distribution and fusion during the welding. The tensile strength of ABS polymer decreases as the traverse speed increases from 10 mm/min to 50 mm/min at both rotational speeds of 800 and 1200 rpm. However, the tensile strength of PC polymer exhibits fluctuations as the traverse speed increases from 10 mm/min to 50 mm/min. This behavior may be attributed to the fluctuating heating and cooling conditions that occur during the welding process at varying rotation and traverse speeds. In contrast to the polymeric base material, the weld zone demonstrated a lower hardness value. The heated tool induces material softening, which results in a reduction in hardness. An examination of alterations in the microstructure of the weld zone was conducted using scanning electron microscopy and stereo microscopy. The observed microstructures were applied to determine the reasons for the decrease in strength. The micrographs illustrate the formation of a fragmentation, attributable to the residual stress generated during the rapid cooling of the liquid polymer. Moreover, a highly increased temperature or traverse speed may result in the formation of voids at the joint interface.

## 1. Introduction

Due to their variety of applications, polymeric materials have grown in popularity in recent years. These materials have many advantages, such as being lightweight, durable, strong, and resistant to corrosion or chemical damage [1]. These qualities have led to a rise in the automotive industry’s usage of polymers. These materials are solid at ambient temperature, but when heated to a specific temperature, they become leathery, followed by a rubbery state or liquid. They become solid again when cooled. Because polymers melt when heated, polymer welding involves the careful control of temperature. Due to this phase transition, polymer welding is considerably more complex than metallic welding [2]. Polymeric materials have been widely used in several industrial applications due to their good properties, such as low density, good moldability, excellent resistance to corrosion, and low production costs. These materials are substituting metals in some applications, especially in the automobile, naval, and aerospace industries [3].

Friction stir welding (FSW) emerged as an alternative welding method to conventional melting-based welding methods in 1991 as a process for joining metals using a non-consumable rotating tool that softens the material through frictional heat and mechanical stirring [4,5]. Compared to traditional welding processes, FSW has many advantages. Because it is performed below the melting temperature, it eliminates common issues found in fusion welding, such as distortion, shrinkage, porosity, and cracks [6,7]. FSW does not generate fumes or spatter, making it a clean process. Since it only requires a simple, robust tool made of hard material, it is a low-cost process with an extended tool life [8,9]. Overall, FSW can improve the quality of joints while lowering production costs. In FSW, a rotating tool with a pin and a shoulder generates frictional heat and mechanical work at the material joint interface. This process can be used for various materials such as metals, polymers, composites, etc [10,11]. This process was then applied to polymers in 1997 [12]. The temperature causes the material to soften but not melt, allowing a joint to be formed by the action of the tool. The softening of the polymer material occurs because the process is performed below its melting temperature. For the FSW process, the direction of the rotating and traversing motions coincide [13]. Therefore, the advancing side (AS) and retreating side (RS) are defined with reference to the tool position, seen from the top of the stirred material. The stir zone (SZ) is where the pin and shoulder surface are in contact with the workpiece, and they experience friction, plastic deformation, and stirring action. The heat produced in this zone travels through conduction to the nugget (welded zone), the heat-affected zone (HAZ), and the base material. The plasticized material flows around the pin due to the shoulder’s stirring action, forming a weld upon cooling. The benefits of FSW include less energy consumption, a lower chance of thermal distortion, lower void content, and lower filler loss [14].

The greatest challenge in FSW is finding process parameters that provide the required thermal profile and flow characteristics necessary to obtain a weld with acceptable quality [15]. The welded joint’s integrity depends on the process parameters, such as rotational speed, traverse (welding) speed, tool geometry, and tool material. Process parameters must be optimized to achieve a strong joint [16].The tool rotation speed directly influences the weld quality and properties in the friction stir welding of polymeric materials. Higher tool rotation speeds result in better mixing and penetration of the material between the pin and the shoulder, while slower speeds lead to a rise in material temperature. However, values of rotation speed above or below an optimal range can diminish the mechanical properties and produce material degradation due to inadequate heat generation or prolonged overheating [17]. In the case of polymeric materials, this is particularly important, as the heat input during the process comes primarily from the friction generated by the tool. The second most critical parameter in friction stir welding (FSW) is with no doubt the traverse speed. The traverse speed represents how fast the tool moves along the weld line. The traverse speed greatly affects the thermal profile on the weld line during FSW and strongly correlates to the maximum temperature. As the effect of rotation speed is fixed, higher traverse speeds mean that polymer needs to be slightly softer to ensure adequate agitation and eventually degradation [18].

Friction stir welding (FSW) tool geometry has major effects on the heat distribution and on the material flow generated during the process [19]. Tool design is therefore critical to prevent defects and to ensure welded components meet the requirements. The most commonly used tool for FSW of polymers consists of a pin and a shoulder made of tool steel [20]. Tool material needs to have high wear resistance, high melting temperature, and low thermal expansion coefficient. The tool geometry can be adapted according to the base material sample configuration, which can be butt or lap joints. In the case of butt joints, the tool can be designed with a wider pin for thicker plates to avoid excessive heat generation [21]. FSW tool features a rotating pin that plunges into the polymer overlapping joint line and a shoulder that covers it. Pins can feature flat, cylindrical, threaded, tapered conical, or stepped-conical designs; shoulders can feature fixed, threaded, or constant-width-style pin profiles [22]. FSW tools have also been devised to simultaneously weld, cut, and create metal inserts carried out on a metallic substrate [23]. Smaller diameter FSW tools can reduce torque requirements during welding because they necessarily deploy lower forces, which is advantageous when welding sensitive materials, such as temperature- or shear-sensitive polymers. Thus, as far as polymers are concerned, smaller pin tools are advantageous [24].

Acrylonitrile Butadiene Styrene (ABS) and Polycarbonate (PC) are both thermoplastic polymers. Acrylonitrile Butadiene Styrene (ABS) is a thermoplastic polymer, derived from three distinct monomers: acrylonitrile (A), butadiene (B), and styrene (S). ABS has high impact strength, good machinability, good surface finish and good tensile strength. Because of its strength, toughness, rigidity, and excellent resistance to heat and chemicals, ABS is used in various applications such as electronic housings, automotive parts, and consumer appliances [25]. PC is also an engineering thermoplastic with excellent impact resistance, toughness, dimensional stability, and flame retardancy. However, it is sensitive to scratches and crazes. Engineering applications of PC can be seen in automotive parts, safety goggles, and optical discs. Both ABS and PC are usually extrusion-molded as sheets for these applications. ABS is cheaper than PC polymer. ABS polymer softens at 200°C and melts at 220˚C. In industrial applications, it can be used at temperatures below 80°C. Friction stir welding of ABS polymer provides stronger welded joint. Although the joint efficiency is lower than the base polymer but it is higher than most other polymers welded joints [26]. PC polymer has high impact strength, good dimensional stability, good transparency and good toughness. PC polymer softens at 125°C and melts at 280˚C. PC polymer welded joint efficiency using butt joint configuration is 48%. In industries, PC can be used for temperature up to 110°C [27].

Recent studies in the literature demonstrate advancements in the friction stir welding of ABS polymer.

Bagheri et al. [28] utilized a 5 mm thick ABS sheet with a custom-built tool, namely a hot shoe, and discovered that welding at elevated rotating speeds and shoe temperatures, along with a reduced tool travel speed, enhances weld quality and tensile strength. Pirizadeh et al. [29] employed a 5 mm thick ABS sheet with a uniquely constructed self-reacting tool, which effectively eradicated root defects and back slits in the welded components. Results demonstrated that an increase in rotational speed correlates with a reduction in tensile strength. Mendes et al. [30] utilized a 6 mm thick ABS sheet with a conical threaded pin and found that high-quality welds are produced without external heating when the tool’s rotational speed and axial force exceed a specific threshold. Mendes et al. [31] utilized a 6 mm thick ABS sheet with a conical threaded pin, demonstrating the viability of robotic friction stir welding (FSW) of ABS without compromising the mechanical qualities of the welds compared to those generated by a specialized FSW machine. Sadeghian et al. [32] worked with an 8 mm thick ABS sheet with cylindrical and conical pins, yielding optimum weld strengths of 97% and 101%, respectively, in comparison to the tensile strength of the base ABS sheet. Yan et al. [33] utilized a 4 mm thick ABS sheet with a triflute pin, and the joints formed with minimal tool stirring had an ultimate shear strength of 20.7 MPa, or 92.8% of the base material’s strength. Moreover, the triflute pin proved to be preferable in generating joints with greater strength compared to the cylindrical pin. Shishavan et al. [34] used nanoparticles as a reinforcement in a 3.7 mm thick ABS sheet with a cylindrical pin and discovered that the inclusion of nanoparticles enhances the surface hardness of the polymeric samples. Furthermore, it was noted that the samples reinforced using multi-walled carbon nanotubes exhibited the maximum hardness value. Stadler et al. [35] applied a 4 mm thick ABS sheet with a cylindrical tool and observed that when the rotational speed during welding increased, the axial force reduced, however the weld strength increased. As the traverse speed escalates, the axial force increases, although the weld strength reduces. Ayaz et al. [36] worked with a 4 mm thick ABS sheet with a threaded cylindrical tool for friction stir spot welding, demonstrating a 20% enhancement in joint strength relative to the initial welding parameters when compared to the optimal welding parameters. Mishra et al. [37] employed a 2 mm ABS sheet in conjunction with a cylindrical pin tool. Machine learning technique was effectively implemented during the FSSW of ABS-ABS sheets to predict UTS and % elongation. The current study shown that the heat input and processing temperature for Friction Stir Spot Welding (FSSW) of ABS sheets may be optimized by reducing spindle speed and dwell time while increasing plunge depth to attain maximum weld strength as well as improved weld quality. Arif et al. [38] utilized a 6 mm ABS sheet with a cylindrical pin profile, and demonstrated that the friction stir welding (FSW) joint from Experiment No. 13 achieved a relative tensile strength of 60%, reaching an ultimate strength of 19.4 MPa, in contrast to the base material’s strength of 32.3 MPa.

Literature contains papers demonstrating recent advancements in the friction stir welding of PC polymer. Derazkola et al. [39] employed a 4 mm thick PC sheet with a frustum pin profile and discovered that the maximum tensile strength and flexural strength was 55 MPa and 61 MPa respectively with a moderate heat input, specifically at 2200 rpm and 105 mm/min. Lambiase et al. [40] utilized a 3 mm thick PC sheet with a threaded pin and observed that elevated welding speeds resulted in increased stresses and temperatures within the weld seam. This occurred with the development of a built-up edge beneath the tool shoulder. The built-up edge caused a milling effect along the weld path, resulting in considerable material removal from the weld joint. Derazkola et al. [41]worked with a 4 mm thick PC sheet with a frustum pin profile to compare Underwater Friction Stir Welding (UFSW) and Friction Stir Welding (FSW), revealing that the maximum tensile strength of the UFSW specimen is approximately 8% more than that of the FSW. Sahu et al. [42] employed a 6 mm thick PC sheet with a conventional pin profile, revealing that the maximum joint efficiency reached 37.01% with a welding configuration of 1800 rpm and 20 mm/min. Derazkola et al. [43] applied a 4mm thick PC sheet with a frustum pin profile and revealed that defect formation during friction stir welding (FSW) of the polymer was likely due to the adhesion of plasticized polymers to the tool and volumetric changes during thermal cycling. Imtiaz et al. [44] employed a 4 mm thick PC sheet utilizing four distinct tools: simple cylindrical conical, threaded cylindrical, threaded conical, and threaded cylindrical conical. The final results indicated that a maximum tensile strength of 51.299 MPa was attained at a traverse speed of 14 mm/min, a rotating speed of 1700 RPM, and utilizing a basic cylindrical conical tool shape. Sahu et al. [45] utilized a 6 mm thick PC sheet with square, cylindrical and triangular pins, determining that the optimal combination for highest joint strength efficiency of 60.06% was achieved at a tool rotational speed of 1800 rpm and a welding speed of 20 mm/min, utilizing a square tool pin profile. Goswami et al. [46] employed a 3 mm thick PC sheet with square, cylindrical, and hexagonal configurations, concluding that the hexagonal tool pin exhibited superior stability in weld profile, a successful uniform weld, the maximum joint strength efficiency (75.4%), and enhanced joint ductility, particularly at elevated tool rotational speeds (2400 rpm). Lambiase et al. [47] employed a 3 mm thick PC sheet with a threaded pin, revealing that welds generated at low welding speeds exhibited weak adhesive between the SZ and the substrate. The welds generated at elevated speeds experienced significant thinning. This led to failure in the SZ region or, in certain cases, within the base material as a result of localized thinning. Dias et al. [48] utilized a 3 mm thick PC sheet with cylindrical and conical pins to determine that the conical pin profile obtained the highest ultimate tensile strength of 34 MPa.

The friction stir welding of ABS and PC polymers has been emphasized by a variety of researchers. Friction stir welding of ABS and PC of less than 5 mm has also been the subject of a limited number of studies. However, there is a lack of sufficient studies in this field. Therefore, this study aims to investigate the weldability of 3 mm thick thermoplastic polymers (ABS and PC). The scope of the study includes the weldability of Acrylonitrile Butadiene Styrene (ABS) and Polycarbonate (PC) by Friction Stir Welding (FSW). The tensile strength of welded polymers will be evaluated using a universal testing machine. The Shore D hardness tester will be used to assess the hardness of the welded polymers. The morphology of joints will also be examined using stereo microscopy and scanning electron microscopy (SEM).

## 2. Materials and method

Following are the materials and methodology used in the friction stir welding of ABS and PC polymers.

### 2.1. Experimentation process

**Fig 1:**
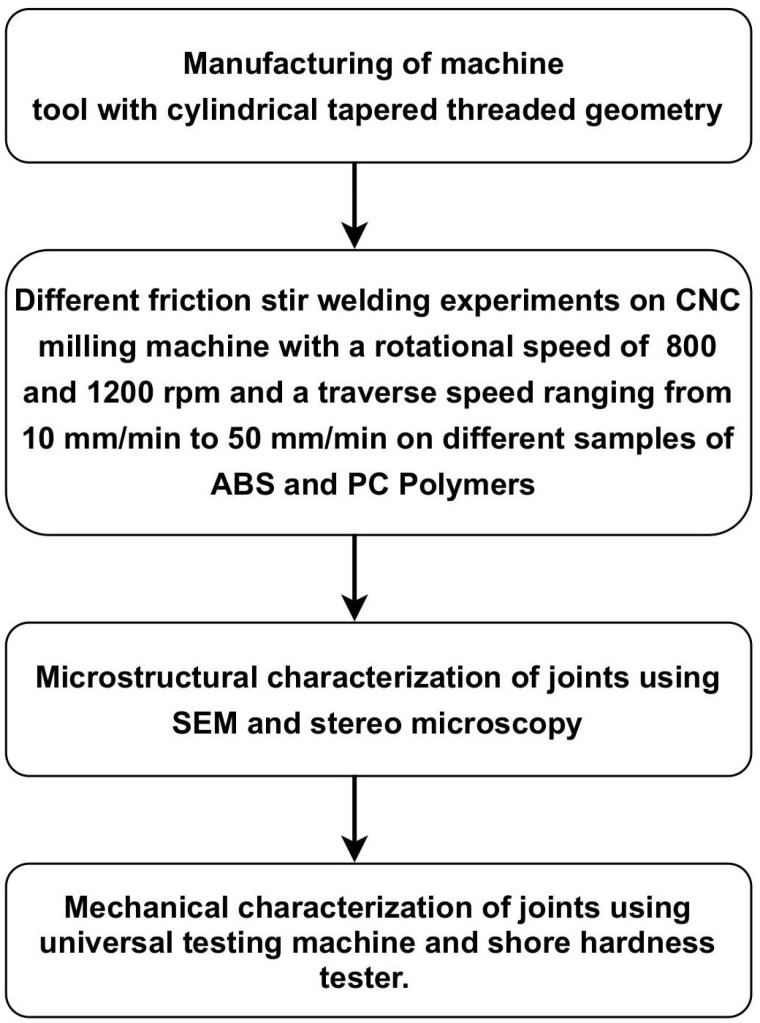
Experimentation plan.

A brief experimentation plan of the complete setup is shown in Fig 1.

### 2.2. Design of tool

The tool, a multidimensional component that comprises a pin and a shoulder, is the primary component of friction stir welding (FSW). Each component is essential to the welding process. The shoulder, through its frictional contact with the workpiece, generates the thermal energy required to soften the material. Concurrently, the pin, acting as a mechanical agitator, propels and mixes the softened material, forming a solid-state weld. The tool’s configuration profoundly influences the weld’s metallurgical characteristics, including grain structure refinement, microstructure homogeneity, and material flow dynamics. The weld nugget area is directly influenced by process parameters and physical elements such as tool geometry. Pin profiles of tapered threaded and tapered cylindrical configuration are recommended for enhanced weld joint strength [49]. It was demonstrated that connections created using threaded profiles exhibited less defects, whereas those formed by non-threaded probes encountered issues. In FSW, the generation of heat and material flow are classified into three categories: inadequate, balanced, and excessive states. The preservation of these states is considerably affected by the tool probe profile. Utilizing the appropriate probe profile enables the production of high-quality, flawless joints. The smooth, featureless surface of the straight, tapered cylindrical probe minimizes friction on the softened material; nonetheless, the application of the tapered, threaded feature results in a successful weld bead. The material is significantly distorted due to the heightened friction of the threaded profile against the softened substance. Therefore, to attain the necessary heat and material flow in the threaded probe profile, resulting in a less defect joints, the levels of heat input and plastic deformation must be appropriate. A sound joint is formed by a tapered threaded probe profile that generates sufficient variable layers.

**Fig 2:**
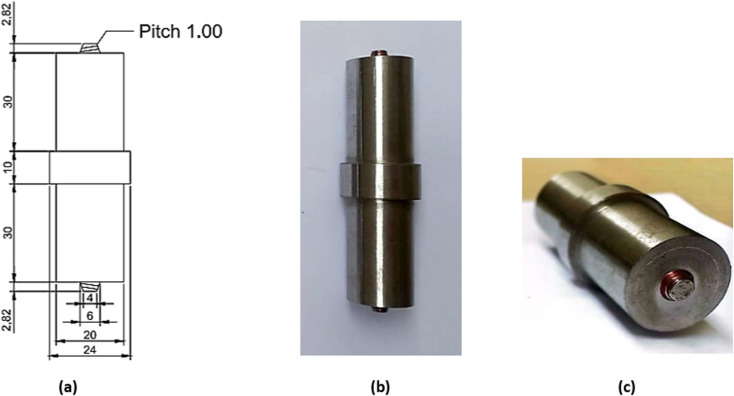
(a) 2D CAD model of tool (b) fabricated tool (c) tool pin geometry.

The tool, containing the collar and shoulder is produced using H13 tool steel rod. Tool of cylindrical tapered threaded has been manufactured as shown in Fig 2 to produce sustainable weld joints. The dimensions of the FSW tool are listed in Table 1. Vacuum heat treatment has been chosen as the method for tool hardening, in accordance with the heat treatment cycle. After tempering, the tools attain a hardness range of 52–57 HRC.

**Table 1:** Dimensions of FSW Tool.

Shoulder Feature	
Shoulder Outer Surface	Cylindrical
Shoulder End Surface	Flat
End Surface Feature	Feature less
**Pin Feature**	
Pin End Surface	Flat
Pin Outer Surface	Cylindrical tapered threaded
**Dimensions**	
Total length of the tool	75.64 mm
Length of both shoulders	30 mm
Diameter of both shoulders	20 mm
Top and Bottom Pin lengths	2.82 mm

### 2.3. FSW experimental setup on modified CNC milling machine

**Fig 3:**
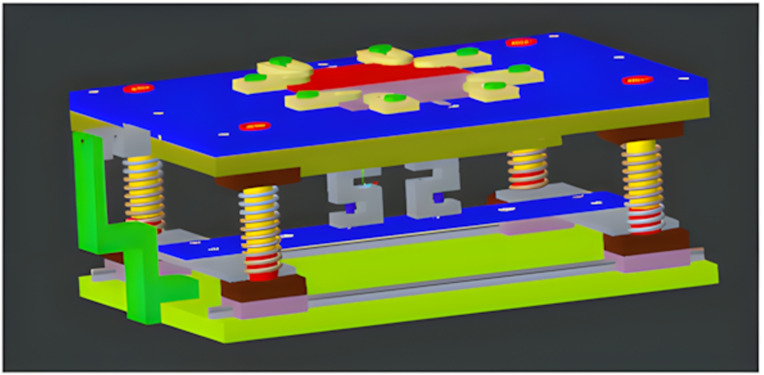
Schematic of fixture.

**Fig 4:**
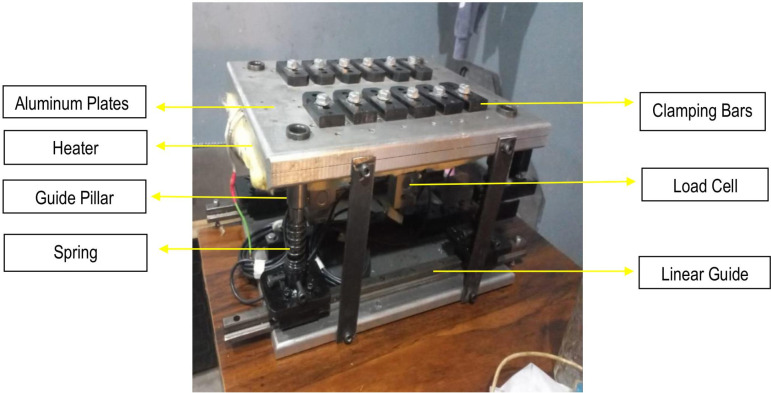
Fabricated Fixture with labelled view.

CNC Vertical Milling machine of Bridgeport (USA) Interact Series is used for FSW with collet size according to the tool diameter and dimensions. Figs 3 and 4, respectively, show the design and fabrication of a fixture for the friction stir welding of ABS and PC polymeric sheets. Fixture is placed on milling machine’s bed which also provide support and sufficient force to the work piece during welding. To use a CNC milling machine for FSW operations, the workpieces must be held firmly by a fixture. This is to make sure that there are no gaps between the plates during clamping, tool impingement, and welding. It should also be safe, easy to use, and take less time to set up. The created fixture is very helpful for doing FSW operations on a CNC milling machine. The fixture is comprised of components, each of which is individually fabricated with the features necessary to address the welding requirements. Fig 4 depicts the various components of the fixture: top plate, bottom plate, back plate, and clamp. It is comprised of 17 mm thick aluminium plates, which serve as backing plates in fixtures owing to their excellent thermal conductivity, compatibility with polymers due to low load application, lightweight composition, thermal stability, and cost-effectiveness. It is appropriate for welding soft materials, such as thermoplastic polymers. The top plate has openings for sliding the clamps according to the size of the to-be-welded workpiece. Other components includes Linear Guides, Guide Pillars with bush, Springs, Load Cells, Clamps, Square bar for fixing load cells, and nut bolts as shown in Fig 4. The main job of the backing plate is to hold the workpiece. During welding, the tool pushes down on plasticized material. A backing plate will maintain the plasticized material within the welding zone. It also protects the top plate in the event that the tool point exceeds the thickness of the workpiece.

**Fig 5:**
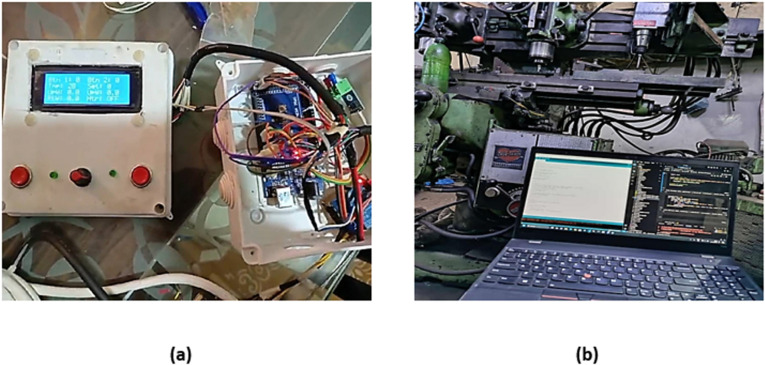
(a) Data Acquisition System (b) Real time data collection.

DAS is a combination of hardware and software that facilitates the measurement or control of physical characteristics to achieve desired outcomes. It consists mostly of three components: signal conditioning, an analog-to-digital converter, and sensors, where data production and collection occur. Data produced by machines were collected in a physical system and transmitted as noisy electrical signals to sensors, which were optimized by signal conditioning (filtering and amplifying) to transform such signals into digital format via an analog-to-digital converter, ultimately shown on a monitor [50]. Data acquisition system (DAS) as shown in Fig 5(a) in FSW plays a crucial role in ensuring precise load cell measurements and effective temperature control. It enables real-time monitoring of load cell readings, providing essential feedback on the mechanical aspects of the welding process as shown in Fig 5(b). This allows operators to make informed adjustments to optimize weld performance.

Strain gauge based S-type load cells are installed in the fixture. The real-time output of load cells is obtained using an Arduino Mega 2560-based data collection device. Software such as Arduino IDE and Visual Studio Code has been utilized to design the output for load cell calculations.

### 2.4. FSW experimental details

**Fig 6:**
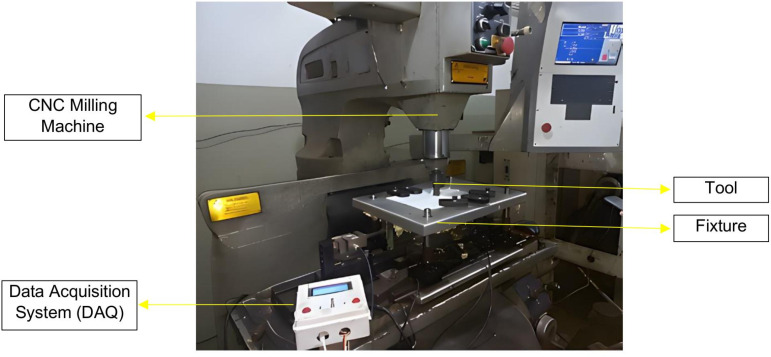
Experimental run of FSW on CNC milling machine.

**Fig 7:**
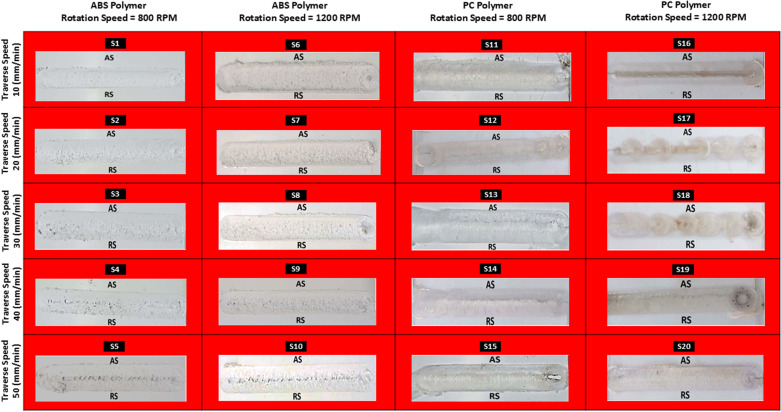
Experimental details.

Acrylonitrile butadiene styrene(ABS) and Polycarbonate (PC) sheets with a thickness of 3 mm were obtained from local suppliers and cut to a size of 150 mm × 100 mm for performing friction stir welding experiments in a butt joint arrangement. A CNC vertical milling machine equipped with a custom fixture and data acquisition system was used for the joining process as shown in Fig 6. The selection of tool rotation speed and traverse speed is a critical aspect for achieving high-quality weld joints. The tool’s rotating speed should be appropriately chosen to ensure adequate heat generation for effective mixing of the weld components in the region of the weld joint [51]. The traverse speed of the tool must be selected to ensure adequate time for material stirring in the weld region of FSW [52]. Iftikhar et al. [49] determined that the optimal parameters for ABS polymer are a tool rotation speed of 650–1100 rpm and a traverse speed of 9 mm/min to 32.5 mm/min. While the optimal parameters for PC polymer are a tool rotation speed of 1220 rpm and a traverse speed of 40 mm/min. However, the parameters are dependent upon the tool profile and the thickness of the polymeric sheets as well. Therefore, prelimary experiments were conducted to define the upper and lower limit of tool rotation speed and traverse speed for a 3 mm thick ABS and PC Polymer. After different pilot experiments were performed, it was found that at tool rotating speeds exceeding 1200 rpm, the weld material heated up and ejected from the weld zone in the partially melted form and sticking to the tool as well. On the other hand, the tool rotational speed is set below 800 rpm, the welded specimen becomes brittle and easily fractured before any subsequent characterization. A non-uniform weld appearance was found when the tool traverse speed was maintained below 10 mm/min. It may be asserted that when the tool traverse speed exceeds 50 mm/min, the weld zone is replete with exterior voids. Following a strategy of trial and error, the range of process parameters was determined for the final experimentation. Fig 7 presents the chosen experimental parameters, including tool RPM and traverse speed. There are two tool rotation speeds (800 and 1200 rpm) and five different traverse speeds (10, 20, 30, 40, and 50 mm/min). The selected parameters led to the welding of 10 ABS samples and 10 PC samples, each with a thickness of 3 mm and with a cylindrical tapered threaded tool.

### 2.5. Characterization of FSW samples

There are a combined total of 20 samples of Friction Stir Welding (FSW), which includes samples made of Acrylonitrile Butadiene Styrene (ABS) and Polycarbonate (PC) polymers. However, those samples exhibiting excellent tensile strengths in (S1, S6, S7, S14, S17) were prepared for stereo microscopy and SEM analysis. The selection is based on the maximum tensile strength of ABS and PC at 800 and 1200 rpm with 10–50 mm/min. The welded polymers will be subjected to ASTM D638-14 standard tensile specimen. The hardness of welded polymers will be subjected to ASTM D2240-05 analysis using Shore D Hardness tester. The morphology of joints will be also analyzed using stereo microscopy and SEM. Table 2 illustrates the techniques used to characterize the various properties of welded joints.

**Table 2:** Different characterization techniques and their uses.

Characterization Technique	Properties of Weld Joints
Stereo Microscope (MOTIC) (DMW-143)	Low resolution surface investigation
Scanning Electron Microscope (JEOL) (JSM 6380A)	Morphological analysis
Shore Hardness Tester (Baxlo Precision)	Shore D hardness
Universal Tensile Testing (Tinius Olsen)	Tensile testing

## 3. Results and discussion

### 3.1. Stereo microscopy of FSW welded ABS and PC polymers

**Fig 8:**
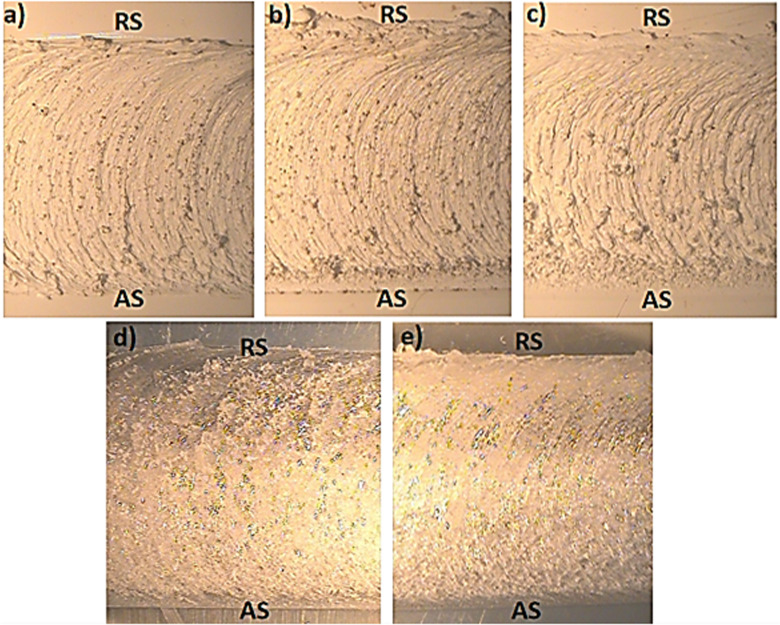
Stereo Microscopy Samples (a) ABS (800 rpm,10 mm/min) (b) ABS (1200 rpm,10 mm/min) (c) ABS (1200 rpm,20 mm/min) (d) PC (800 rpm,40 mm/min) (e) PC (1200 rpm,20 mm/min).

Stereo microscopy is best suited to detailed analysis of surface textures and defects, including inclusions as well as overall uniformity within the weld zone, heat-affected zone, and base material. It allows the observation of fine details while analyzing the structural changes that occur during friction stir processing to produce weld joints [30]. Fig 8(a) shows the stirring in the weld zone is generated by the rotational motion of the tool,; as a result, concentric spiral patterns are generated on the surface of the weld zone. These features are attributed to the effectiveness of the mixing process as well as heat distribution during welding. The surface appears to be homogeneous, which validates that the process parameters were rightly set and effectively controlled during the welding process, resulting in uniform heat distribution in the weld zone. Inclusions such as small particles and bubbles are shown in the weld nugget, which are produced due to trapped air or polymeric debris. This is due to the reason that air is entrapped in the weld zone due to the rotation of the tool, resulting in air voids in the weld nugget. As illustrated in Fig 8(b), the application of a higher rotational speed results in the formation of a well-defined weld bead, which is attributed to the effective flow of materials within the stirring zone, adequate heat input, and a reduced number of surface defects. ABS polymer does not exhibit any indications of thermal degradation, indicating that the welding process is provided sufficiently heat input. The material flow becomes more readily achieved during the welding process as a result of the increased rotation speed, which leads to a consistent and uniform surface. The process was effectively stirred, as evidenced by the visible tool traces. Small particles were present; however, they did not ultimately influence the strength of the weld [31]. As depited in Fig 8(c), the stereo micrograph of the ABS joint that was successfully welded at 1200 rpm with a 20 mm/min speed. The tool’s time spent on the polymer is reduced as a result of the increased traverse speed, which in turn leads to a lower heat input and a lower temperature in the weld zone. Nevertheless, the surface remains relatively smooth despite of the increased rotation speed. Fig 8(d) shows that during the welding process, the polycarbonate polymer endures plastic deformation, which results in effective material stirring. The weld surface appears rough and granular as a result of the heat and mechanical action that occur during the welding process. This roughness may suggest that a more effective stirring process was accomplished; however, excessive roughness could potentially impact the mechanical properties and surface finish. A higher traverse speed of 50 mm/min suggests that the heat input is reduced, and the polymer’s thermal degradation is restricted by the rapid cooling. InFig 8(e), the PC weld joint is demonstrated to be produced at higher rpm and traverse speed. Due to the increased heat input resulting from the increased rotation speed of the tool, effective plasticization and material flow are observed at the polymer-tool interface. In comparison to lower traverse speeds, higher traverse speeds reduce dwell time, which leads to rapid cooling and the prevention of unnecessary thermal degradation [40].

### 3.2. Scanning electron microscope (SEM) of FSW welded ABS and PC polymers

**Fig 9:**
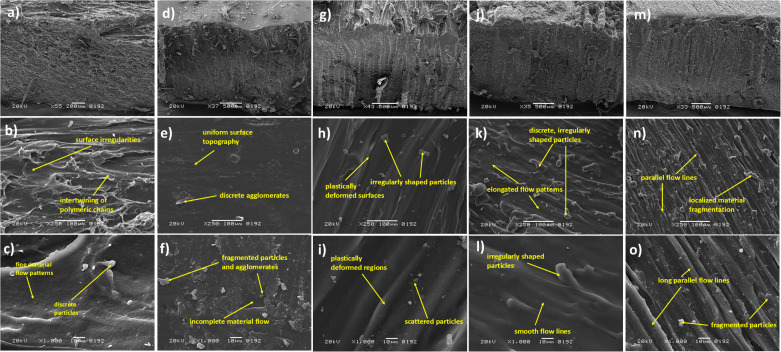
SEM Samples of FSW Joints: (i) ABS (800 rpm,10 mm/min) [(a) Cross-sectional View, (b) 250X, (c)1000X} (ii) ABS (1200 rpm,10 mm/min) [(d) Cross-sectional View, (e) 250X, (f)1000X} (iii) ABS (1200 rpm,20 mm/min) [(g) Cross-sectional View, (h) 250X, (i)1000X} (iv) PC (800 rpm,40 mm/min) [(j) Cross-sectional View, (k) 250X, (l)1000X} (v) PC (1200 rpm,20 mm/min) [(m) Cross-sectional View, (n) 250X, (o)1000X}.

The cross-sectional views of the samples are depicted in Figs 9(a, d, g, j, m). The weld nugget demonstrates the effective polymerization of the polymers with minimal voids resulting from the plasticization of the materials in the weld zone. Figs 9(b,c) shows the irregular surface features indicating the dynamic flow of material and mechanical sittirng of materials that occurs due to deformation of polymer and microstructural changes that occurs during welding. Localized material disintegration appears as fragmented particles or polymer debris due to elevated temperature and shear forces. The intertwining and elongation of ABS polymeric chains result from substantial stirring of the material. Increased magnification reveals that the tool rotation induces plasticized polymer flow and the formation of fibrous structure appears on the surface [28]. Micro-voids are generated during the welding process, as shown by the presence of tiny agglomerates and particles. Figs 9(e,f) illustrate that scattered and irregular particles are observed as a result of fragmentation or thermal degradation of polymer. These particles may arise from the interface between the tool and polymer, where elevated frictional heat and significant shear stresses lead to the rupture of ABS polymeric chains, resulting in the formation of particulates. Localized fragmentation of ABS polymer is distinctly observable at higher magnification, attributable to increased tool rotation speed, which generates enhanced frictional heat and high shear forces. The dispersion of these particles suggests a significant likelihood of localized stress, material instability, and inadequate polymer flow. Figs 9(h,i) illustrate the deformation and redistribution of polymeric chains resulting from increased frictional heat and elevated shear stresses. Irregularly shaped particles are present, resulting from heat deterioration or material fragmentation during the welding process [37]. The increased tool rotation speed generated greater localized shear stresses, resulting in the fragmentation of the polymer. These particles indicate that a localized material instability arises during welding due to increased tool rotation speed. Figs 9(k,l) illustrate the weld nuggets of PC polymer, wherein the surface morphology exhibits fine and elongated flow patterns resulting from plastic deformation and polymer stirring during welding. These properties contribute to the high degree of plasticization and effective stirring [41]. Nonetheless, irregularly shaped particles dispersed around the weld nugget could be attributable to material fragmentation or heat deterioration during the welding process. Intense shear stresses and frictional heat caused localized degradation of the PC polymer [42]. Figs 9(n,o) demonstrate elongated parallel flow lines, demonstrating successful stirring and plastic deformation of the polymer within the weld zone. Small aggregates are dispersed along the parallel flow lines as a result of the localized fragmentation of the polymer. The particles are produced by the contact at the tool polymer interface, resulting from elevated frictional heat and high shear forces that convert the polymeric components into smaller particulates. These particles indicate localized mechanical and thermal stresses resulting from stress-induced deterioration in various regions of the weld nugget [45,47]. During the welding of PC polymer, material thinning occurs due to the development of a built-up edge beneath the tool shoulder. The built-up edge generated a milling effect along the weld direction, resulting in considerable material being removed from the weld joint [40].

### 3.3. Tensile testing and joint efficiency of FSW welded ABS and PC polymers

**Fig 10:**
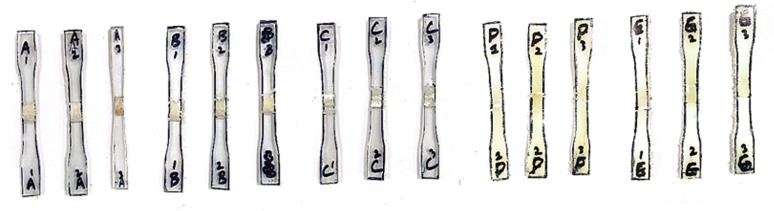
Dog-bone specimens before tensile testing.

**Fig 11:**
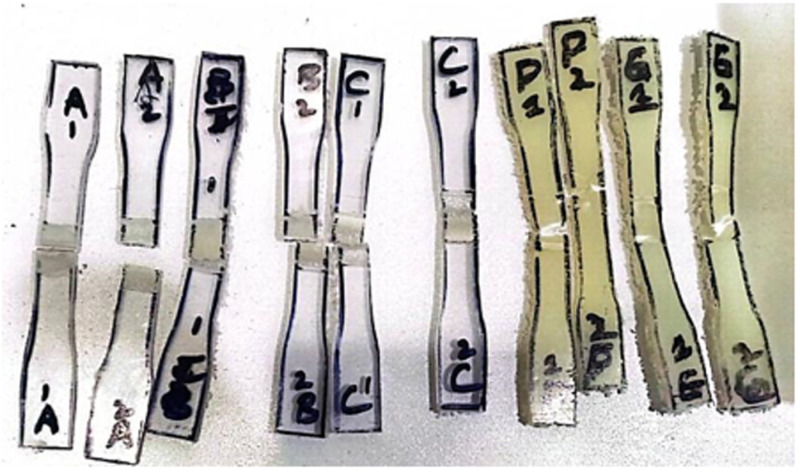
Dog-bone specimens after tensile testing.

The tensile strength of samples were tested using the universal testing machine with the specimen prepared according to the ASTM D638-14 standard as shown in Figs 10 and 11. The tensile strength of the ABS polymer base material is 38.70 MPa, whereas that of the PC polymer is 60 MPa.

**Fig 12:**
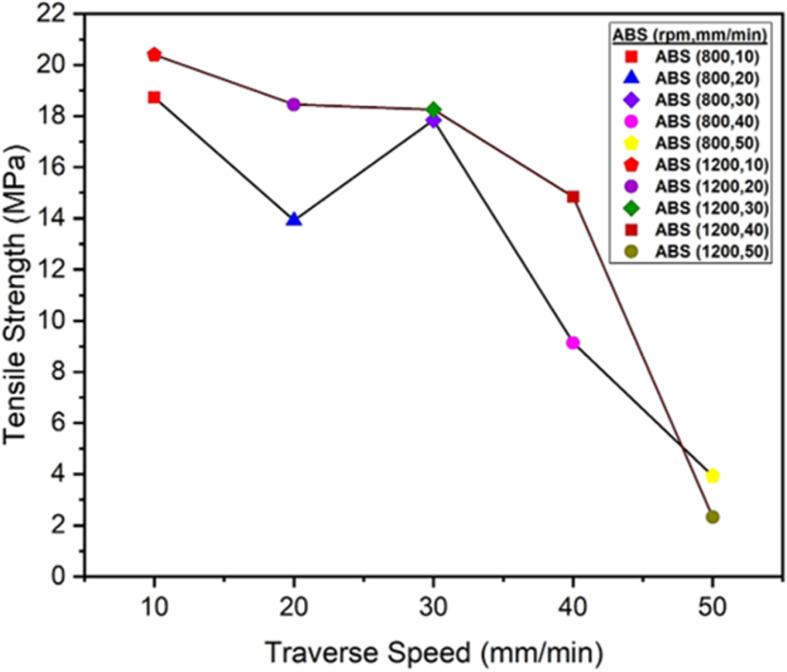
Tensile Strength (MPa) of ABS Polymers at 800 and 1200 rpm.

The graph at Fig 12 shows how the tensile strength of an ABS polymer changes when friction stir welding is used at different traverse speeds (10–50 mm/min). The tensile strength exhibits a downward trend at both rotational speeds of 800 and 1200 rpm when the traverse speed increases from 10 mm/min to 50 mm/min. At 800 rpm, the maximum tensile strength at 10 mm/min is 18.73 MPa, and the minimum tensile strength at 50 mm/min is 3.94 MPa. At 1200 rpm, the maximum tensile strength at 10 mm/min is 20.40 MPa, and the minimum tensile strength at 50 mm/min is 2.33 MPa. This indicates that higher traverse speeds result in inadequate joint strength due to insufficient time for heat dissipation and proper bonding of ABS polymer [30]. Loss of tensile strength is caused by changes in the ABS polymer on a microscopic level, such as poor bonding and the formation of defects like voids and porosity [31]. Lower traverse speed supports the improvement of strength because of effective heat distribution and enables effective material fusion during the welding process. It also indicates that the material moves through a higher level of intense fusion phase, causing a higher level of strength; however, it could potentially open localized weak points as well. Furthermore, a lower traverse speed seems to suggest higher tensile strength. This advantage could be because it manages heat better and bonds materials better during the welding process. Additionally, it could impact material properties in unlikely or weakly defined regions of welded areas due to excessively high speeds [32].

**Fig 13:**
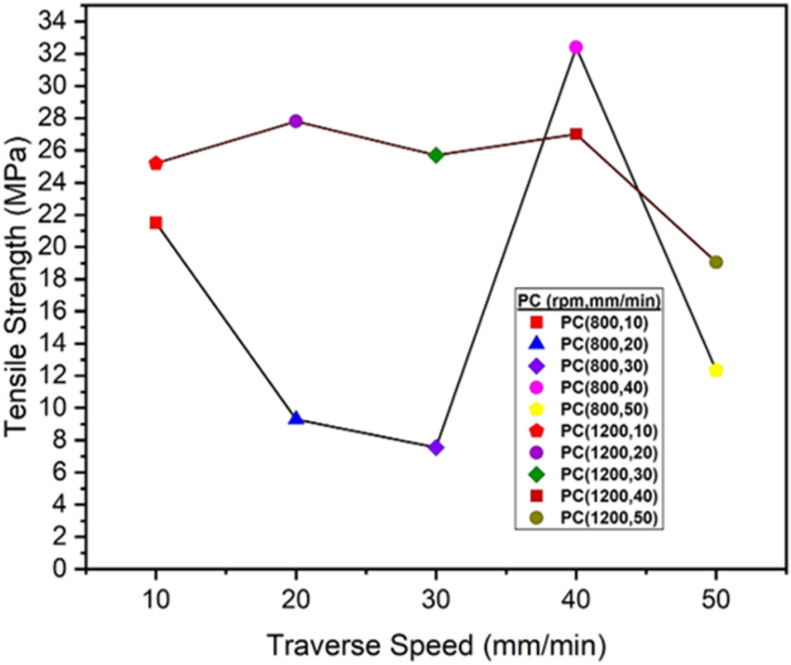
Tensile Strength (MPa) of PC Polymers at 800 rpm and 1200 rpm.

The graph at Fig 13 shows how the tensile strength of an PC polymer changes when friction stir welding is used at different traverse speeds (10–50 mm/min). The tensile strength demonstrates a declining trend at rotational speeds of 800 rpm from 10 mm/min to 30 mm/min, peaking at 40 mm/min before decreasing again. Conversely, at 1200 rpm, the tensile strength shows fluctuations, decreasing and then increasing as the traverse speed rises from 10 mm/min to 50 mm/min. At 800 rpm, the maximum tensile strength at 40 mm/min is 32.40 MPa, and the minimum tensile strength at 30 mm/min is 7.56 MPa. At 1200 rpm, the maximum tensile strength at 20 mm/min is 27.80 MPa, and the minimum tensile strength at 50 mm/min is 19.05 MPa. The weld quality was inadequate at low tool rotating speeds combined with high traverse speeds due to inappropriate heat input [45]. The graph clearly indicates that increased tensile strength is attained at low rotational speeds and high traverse speeds. This behavior occurs because low rotational and traverse speed generate less heat, which prevents appropriate mixing of the materials from the two adjacent surfaces, resulting in poor weld quality [44]. At increased rotational and traverse speeds, the material melts rapidly due to increased heat generation; however, these higher speeds prevent adequate settling of the material, resulting in porosity inside the weld joint [47,48]. Zafar et al. [53] indicated that welding parameters differ among various polymers. Materials with elevated melting points and viscosities necessitate increased rotational speeds and reduced traversal speeds to attain adequate heat and optimal joint strength.

**Fig 14:**
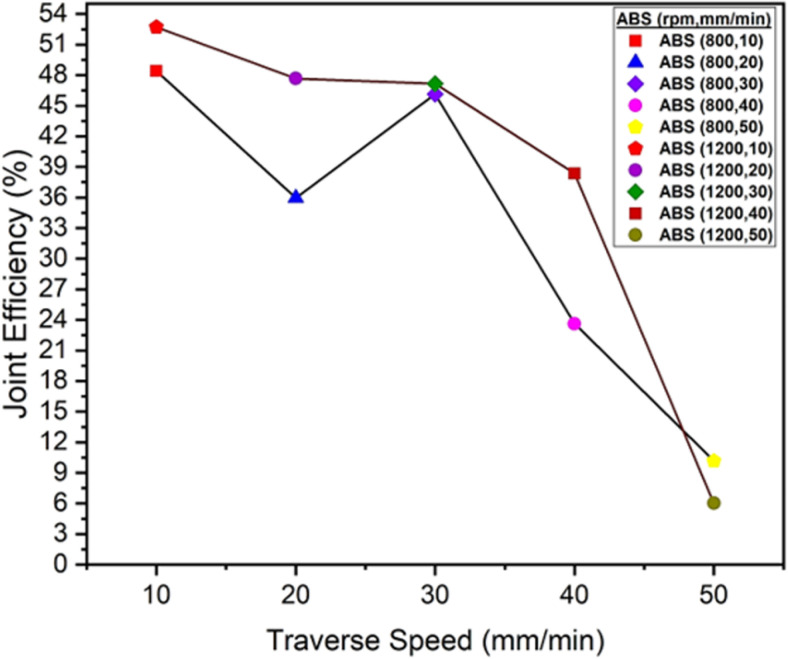
Joint Efficiency (%) of ABS Polymers at 800 rpm and 1200 rpm.

Fig 14 illustrates the variation in percent joint efficiency of an ABS polymer when subjected to friction stir welding at various traverse speeds ranging from 10 to 50 mm/min. The joint efficiency demonstrates a declining trend at both rotational speeds of 800 and 1200 rpm when the traverse speed increases from 10 mm/min to 50 mm/min. At 800 rpm, the maximum joint efficiency at 10 mm/min is 48.40%, and the minimum joint efficiency at 50 mm/min is 10.18%. At 1200 rpm, the maximum joint efficiency at 10 mm/min is 52.71%, and the minimum joint efficiency at 50 mm/min is 6.02%. This indicates that increased traverse speeds lead to weaker joints due to inadequate heat generation and material mixing during welding. It also shows that a low traverse speed supports the improvement of joint strength because of an effective heat distribution during welding process.

**Fig 15:**
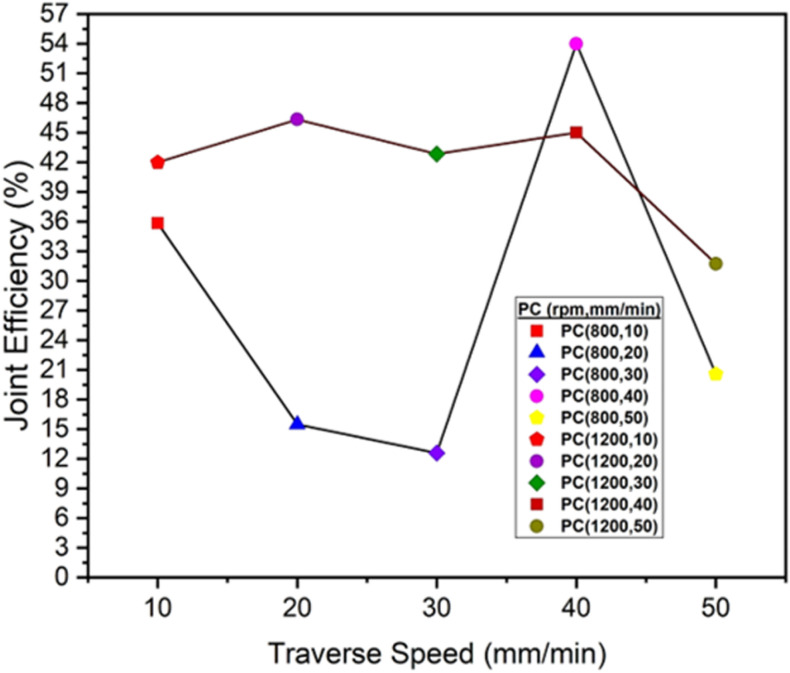
Joint Efficiency (%) of PC Polymers at 800 rpm and 1200 rpm.

Fig 15 illustrates the variation in percent joint efficiency of an PC polymer when subjected to friction stir welding at various traverse speeds ranging from 10 to 50 mm/min. The joint efficiency demonstrates a declining trend at rotational speeds of 800 rpm from 10 mm/min to 30 mm/min, peaking at 40 mm/min before decreasing again. Conversely, at 1200 rpm, the joint efficiency shows fluctuations, decreasing and then increasing as the traverse speed rises from 10 mm/min to 50 mm/min. At 800 rpm, the maximum joint efficiency at 40 mm/min is 54%, and the minimum joint efficiency at 30 mm/min is 12.60%. At 1200 rpm, the maximum joint efficiency at 20 mm/min is 46.33%, and the minimum joint efficiency at 50 mm/min is 31.75%. The decrease in joint efficiency at 800 rpm suggests that the slower traverse speed might not be able to support enough heat input, leading to weaker welding and strength. At 1200 rpm, higher traverse speed allows for effective heat distribution and fusion, leading to significant enhancements in joint efficiency of PC polymer. However, a slight variation in the joint efficiency of PC polymer potentially occurring in specific welded regions due to fluctuating heating and cooling conditions during the welding process at different rotation and traverse speeds. Table 3 provides a brief overview of the tensile strength and joint efficiency of ABS and PC welded samples.

**Table 3:** Tensile strength and joint efficiency of ABS and PC welded samples.

Sample No.	Material	Rotation Speed (RPM)	Traverse Speed (mm/min)	Tensile Strength (MPa)	Joint Efficiency (%)
1	ABS	800	10	18.73	48.40
2	ABS	800	20	13.92	35.97
3	ABS	800	30	17.85	46.12
4	ABS	800	40	9.14	23.62
5	ABS	800	50	3.94	10.18
6	ABS	1200	10	20.40	52.71
7	ABS	1200	20	18.45	47.67
8	ABS	1200	30	18.25	47.16
9	ABS	1200	40	14.84	38.35
10	ABS	1200	50	2.33	6.02
11	PC	800	10	21.50	35.83
12	PC	800	20	9.29	15.48
13	PC	800	30	7.56	12.60
14	PC	800	40	32.40	54.00
15	PC	800	50	12.35	20.58
16	PC	1200	10	25.20	42.00
17	PC	1200	20	27.80	46.33
18	PC	1200	30	25.70	42.83
19	PC	1200	40	27.00	45.00
20	PC	1200	50	19.05	31.75

### 3.4. Shore hardness test of FSW welded ABS and PC polymers

The specimens were prepared in accordance with the ASTM D2240-05 standard, and the shore hardness of the samples was assessed using a shore hardness tester. The shore hardness of the ABS polymer base material is 78 MPa, whereas that of the PC polymer is 87 MPa.

**Fig 16:**
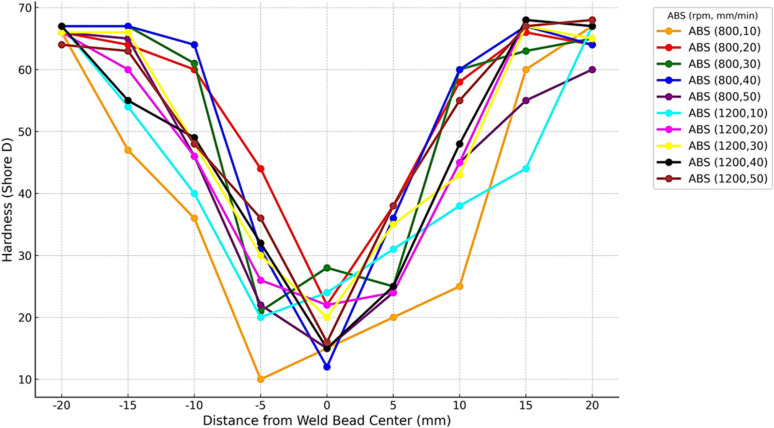
Hardness Profile of ABS Polymers at 800 and 1200 rpm.

The graph in Fig 16 shows the hardness profile of ABS polymer. It indicates the change of hardness values at weld bead ranging from -20 mm to + 20 mm, which comprises of key regions including stirring zone/ thermo-mechanically affected zone (TMAZ), and heat affected zone. It can be observed that more heat is transferred towards the material at lower traverse speeds, leading to a higher level of hardness in weld bead and HAZ respectively. Also, the tool continues to be in contact with material for a longer duration of time which ultimately increases the temperature and mechanical work across the region [54]. Moreover, the tool advances quickly at higher traverse speeds indicates a lower heat input and a decrease in the impact of material. It leads to a lesser hardness value in the stirring zone and HAZ due to limited availability of heat to influence the most important changes on microstructure to improve hardness [55].

**Fig 17:**
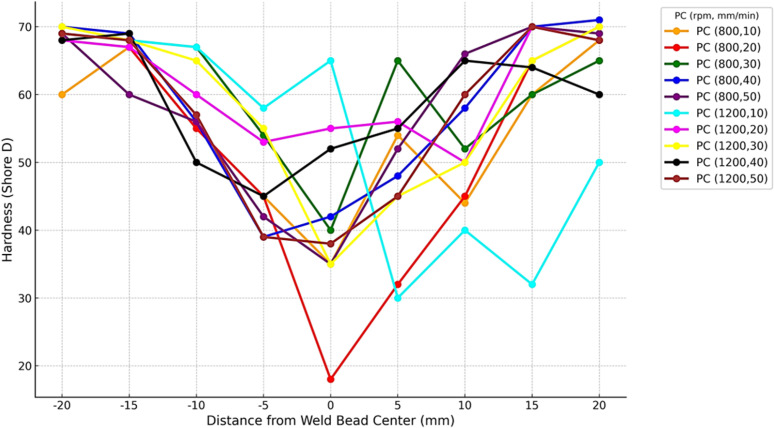
Hardness Profile of PC Polymers at 800 and 1200 rpm.

The graph in Fig 17 shows the hardness profile of PC polymer. It indicates the change of hardness values at weld bead ranging from -20 mm to + 20 mm, which comprises of key regions including stirring zone/ thermo-mechanically affected zone (TMAZ), and heat affected zone. It can be seen from the graph that higher hardness values are usually found in samples with lower traverse speeds because of the increase in heat input. However, those samples which have higher traverse speed shows decline in hardness in such regions because of decrease in heat input. The weld zone exhibited a lower hardness value in comparison to the the base materials [39]. The decrease in hardness is a result of material softening caused by the heated tool [41].

ABS and PC both polymers show similar trends relative to hardness variation with distance from weld bead, however PC polymer continuously indicates higher hardness in comparison to ABS polymer. The ABS weld joints has lower hardness than those of the PC weld joints as shown in Table 4. The possible reason may be that the viscous dissipation of material with a higher flow temperature will release more heat compared to that with a lower flow temperature. Another possible reason is that the material with a higher flow temperature tends to deform into a thicker bond line, causing a smaller grain size compared to that of a thinner bond line, during the welding process [42,45]. Table 4 shows average shore hardness values of ABS and PC welded samples.

**Table 4:** Average shore hardness values of ABS and PC welded samples.

Sample No.	Polymer	RPM	Traverse Speed (mm/min)	Average Shore Hardness (“D” scale) Heat Affected Zone	Average Shore Hardness (“D” scale) Weld Stirring Zone
1	ABS	800	10	60	21.2
2	ABS	800	20	65	44.4
3	ABS	800	30	65.5	39
4	ABS	800	40	66.25	40.6
5	ABS	800	50	61.5	30.4
6	ABS	1200	10	58	30.6
7	ABS	1200	20	65.25	32.6
8	ABS	1200	30	66	35.2
9	ABS	1200	40	64.25	33.8
10	ABS	1200	50	65.5	38.6
11	PC	800	10	63.75	46.6
12	PC	800	20	67.5	39
13	PC	800	30	65.5	55.6
14	PC	800	40	70	48.6
15	PC	800	50	67	50.2
16	PC	1200	10	54.75	52
17	PC	1200	20	68.25	54.8
18	PC	1200	30	68.25	50
19	PC	1200	40	65.25	53.4
20	PC	1200	50	68.75	47.8

### 3.5. Axial load calculations of FSW welded ABS and PC polymers

**Fig 18:**
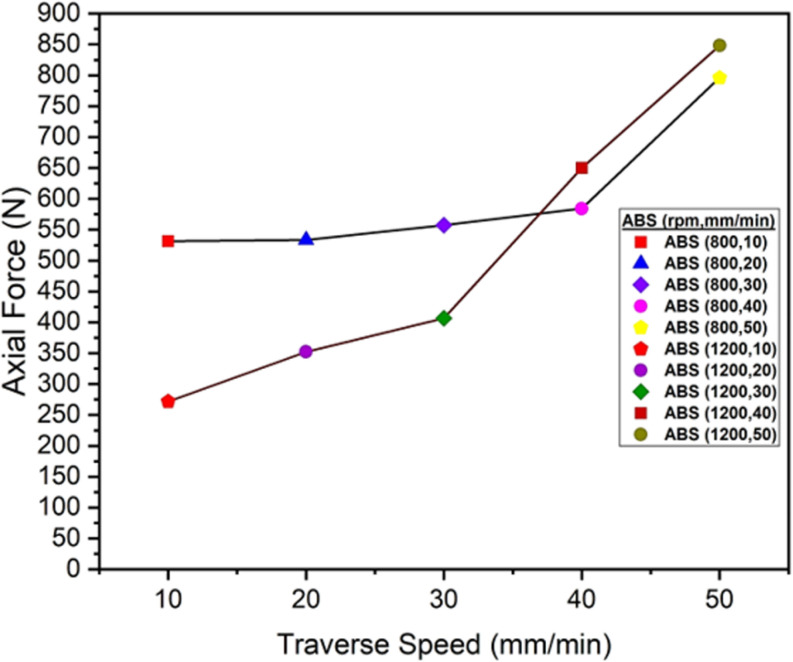
Maximum Axial Force values of ABS Polymers at 800 and 1200 rpm.

**Fig 19:**
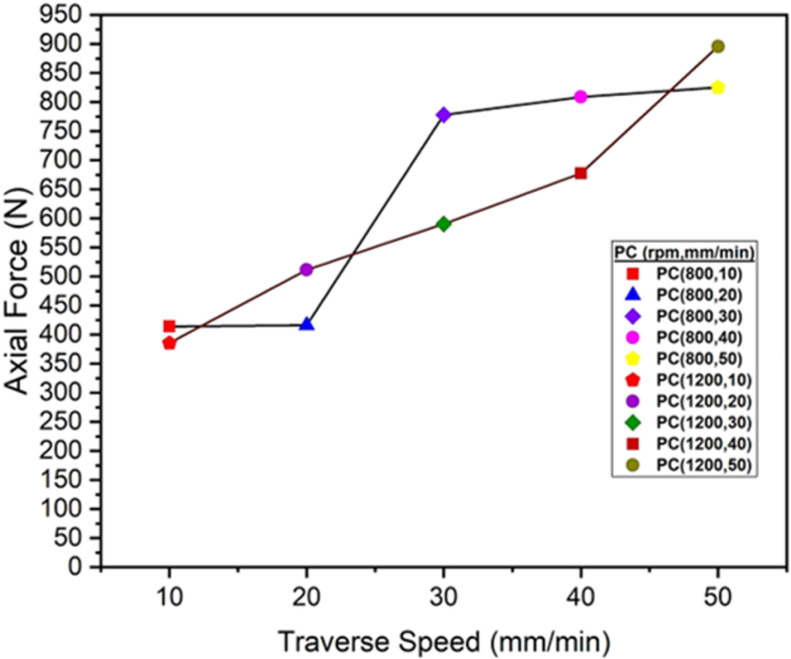
Maximum Axial Force values of PC Polymers at 800 and 1200 rpm.

The axial force, a downward or vertical force, is applied by the plunging force of the tool into the ABS and PC polymers. The evaluation of these values are shown in Fig 18 and Fig 19.

The graph at Fig 18 and Fig 19 represents the maximum axial force (N) during the friction stir welding of ABS and PC polymers at rotational speeds of 800 and 1200 rpm. The traverse speed ranges from 10 mm/min to 50 mm/min. In the tool plunging stage, there is a material softening in which, for all traverse speeds, the axial force usually begins at a low value and then gradually increases over time. As the tool moves forward, there is an increasing trend in the axial force, and then the force becomes steady over the period of time. Due to the increased in material resistance, there is a greater axial force experienced in higher traverse speeds, i.e., 50 mm/min. Extremely high tool rotation speeds, however, cause excessive material deterioration and incorrect tool penetration. The rate at which the tool moves along the weld line corresponds to traverse speeds. There is less time available for the interaction of the tool with the material and the generation of heat at higher traverse speeds, such as 40 mm/min or 50 mm/min. As a result, the tool requires more force to maintain the plasticization of the material, leading to an increase in the axial force. On the other hand, lower traverse speeds, such as 10 mm/min or 20 mm/min, exhibit more time for the material to generate heat and become malleable, which results in the decrease in axial load. There are different factors on which axial force depends, such as traverse speeds, tool rotation speed, and mechanical and thermal properties of materials [56]. Higher traverse speeds shows higher axial force because the material experiences more resistance to deformation which leads to greater axial force at higher rate of traverse speeds [46]. In conclusion, the axial force decreases with increasing RPM, whereas the axial force increases with increasing traverse speed. Higher tool rotation speeds, such as 1200 rpm, increase the heat generation because of the intense frictional interaction with the material. As a result, this process leads to the localized softening of the material and the reduction of forces experienced at lower traverse speeds [35].

## 4. Conclusion

This study investigates friction stir welding with a cylindrical tapered threaded tool on 3 mm thick ABS and PC polymers, altering the tool rotational speed (800 and 1200 rpm) and tool traverse speed (10 mm/min to 50 mm/min). Following conclusions can be drawn based on the experimental results achieved.

- For ABS polymer at 800 rpm, the highest tensile strength at 10 mm/min is 18.73 MPa, whereas the lowest tensile strength at 50 mm/min is 3.94 MPa. At 1200 rpm, the highest tensile strength at 10 mm/min is 20.40 MPa, while the lowest tensile strength at 50 mm/min is 2.33 MPa.
- The highest tensile strength of PC polymer at 800 rpm is 32.40 MPa at a traverse speed of 40 mm/min, whereas the minimum tensile strength is 7.56 MPa at 30 mm/min. At 1200 rpm, the maximum tensile strength at 20 mm/min is 27.80 MPa, while the minimum tensile strength at 50 mm/min is 19.05 MPa.
- For ABS, the maximum joint efficiency achieved is 52.71% with a rotational speed of 1200 RPM and a traverse speed of 10 mm/min.
- For PC, the maximum joint efficiency is 54% with a rotational speed of 800 RPM and a traverse speed of 40 mm/min.
- For ABS and PC polymers, the axial force decreases with increasing RPM, whereas the axial force increases with increasing traverse speed. Higher tool rotation speeds, such as 1200 rpm, increase the heat generation because of the intense frictional interaction with the material. As a result, this process leads to the localized softening of the material and the reduction of forces experienced at lower traverse speeds.
- In terms of hardness variation with distance from the weld bead, ABS and PC polymers exhibit comparable behaviors. However, PC polymer exhibits a higher hardness than ABS polymer.
- The weldability of Polycarbonate (PC) and Acrylonitrile butadiene styrene (ABS) with a thickness of 3 mm has substantial potential. Nevertheless, additional research is necessary to thoroughly investigate this potential. Welds with a reasonable level of tensile strength are produced by friction stir welding (FSW). The melt viscosity of the polymers may be the reason for the substantial variation in the optimal joining parameters among both polymers. These aspects necessitate further investigation.
